# Dentists and the American Medical Association

**Published:** 1892-01

**Authors:** 


### DENTISTS AND THE AMERICAN MEDICAL ASSOCIATION.

THE last number of this journal (December) contained a circular letter from Dr. Dunglison, secretary of the American Medical Association, and an accompanying explanatory letter from Dr. Talbot, of Chicago, ex-president of the Oral Section of that organization.

The notice of Dr. Dunglison was remarkable for the absence of any allusion to dentists or the resolution adopted by the American Medical Association, and, as it stands, has no force with those holding the D.D.S. degree. There has been a constant misunderstanding in relation to the position occupied by dentists in this body, which is explained in the letter of Dr. Talbot, and there need be no further misconception in regard to the position occupied by those who hold the so-called "partial degree."

That this explanation will be satisfactory is exceedingly doubtful. It involves so many conditions that it practically makes membership in that organization impossible. The first requirement is that an applicant for membership must belong to an association working under the code of ethics of the American Medical Association. In the second place, he must be a graduate of a department connected with a medical school or university; and third, if so connected, he must have taken the regular course with the medical students, omitting certain studies and replacing these with certain others.

The first condition is possible of attainment, provided a sufficient number of members of a local society should be willing to adopt the code required. Membership in medical local societies is impossible, at least in the East, for those holding the D.D.S. degree. This being so, we cannot understand how a dental society can be recognized as a whole; indeed, it seems directly in conflict with the constitution, which requires membership in medical societies.

The matter is so much mixed and so inconsistent with itself that explanation serves only to make confusion worse confounded.

If the society or club adopting the code can be represented, there will certainly be delegates sent and application for membership made by persons who have not complied with the second requirement by graduating from a department of a medical college or university.

It must be recognized that this open door, if it means all that Dr. Talbot claims for it, is a liberal construction of the constitution, and as such should meet with a generous response.

The third requirement means the rejection of all those not graduates of certain schools, or, in other words, it says emphatically to these schools, "Your graduates can never be recognized by medical men as specialists in medicine."

The question of recognition is not one that interests us, and, further, it has been amply discussed elsewhere. The only thing to be considered is, is it just? We cannot so regard it.

The degree of M.D. in the United States has been trailed in the mire from the first organization of medical schools. It should stand for the highest culture in this profession. Does it do this? Until within the last three years nearly three-fourths of all medical schools in this country had a course of two years, or two sessions of generally five months each. The report of the Commissioners of Education for 1885–86 gives the number of regular medical schools as ninety-four; of these, seventy-three (73) had courses of from one to two years; twenty-one (21), from three to four years. The report for 1887–88, the last issued, gives the number of the regular schools in the United States as eightynine; of these, twenty-four (24) had two years, and sixty-five (65) had courses of from three to four years. Of this number in 1887–88, fifty-two (52) had courses of from five to six months, nineteen (19), over six months, and eighteen (18) failed to report.

It will thus be seen that up to the period of 1885–86 the M.D. degree was conferred in a large majority of the schools after two courses of about five months each, and even at the date of the last report there were still a large number graduating men in the same period. It cannot be denied that men have been sent out by the thousands, to stand by the bedsides of other thousands in their last extremity, with an amount of theoretical information so limited that it is not surprising that to the average layman the titles doctor and undertaker are nearly synonymous.

While dental colleges have been equally derelict in their duty in the past, there can be no comparisons drawn between the two conditions when the amount of work and responsibility in medicine is considered.

It must, therefore, be recognized that, however exalted the M.D. degree may be in the estimation of society at large, to the specialist it means something or nothing just in proportion to the amount of training and the character of that training.

The dental schools of the country have now, without any exception, three years, the majority with five months as a term. Under this state of things it may not be wholly inappropriate for the dentist of the near future to retort, with some degree of consistency, "We refuse indiscriminate association with men who make the impossible claim of having been educated in medicine in two years."

The main question now is, Is it possible that dentists can, in the present state of affairs, work harmoniously with medical men? No man can or should be recognized, no matter what degree he may hold, unless he is able to demonstrate that he has that above all degrees, knowledge and ability to place his information in practical directions for the good of humanity and for the advancement of science. We hold that a large proportion of dentists have arrived at that condition of culture, and are able to maintain not a mediocre position, but one that must reflect credit on themselves and medical science; for whatever has been acquired in a special study becomes legitimately part of the whole. It is time that dentists should abstain from asking for recognition. It is humiliating!

The appeal, if it can be called such, of Dr. Talbot, for dentists to unite with those bodies of a truly scientific character, should receive earnest attention. The day is past for organizations such as we have had, and the line must be positively drawn between those who join societies for selfish purposes and those who have only the highest professional and scientific interests at heart. We believe this may be attained in an association in dentistry proper. If it exists in the Section of Dental and Oral Surgery of the American Medical Association, it is a positive misfortune to that body that it is deprived of the hearty co-operation of men worthy in every respect, but who cannot show that they graduated from a medical college, but are simply energetic workers in their chosen field of scientific research, with a degree that has not yet received the stamp of respectability from the social world. The highest attainment any one can reach is a self-respecting culture, and this will command recognition everywhere where civilization extends, and is not wholly dependent on parchments or the artificial bars that caste places round the recognized professions.

